# Heating Before or After Complexation Differentially Affects Structural and Functional Properties of Whey Protein Isolate–Gallic Acid Complexes

**DOI:** 10.3390/foods15101714

**Published:** 2026-05-13

**Authors:** Hesti Ayuningtyas Pangastuti, Songsak Wattanachaisaereekul, Supatra Karnjanapratum, Praphan Pinsirodom

**Affiliations:** 1 Food Technology Program, Faculty of Industrial Technology, Institut Teknologi Sumatera, Terusan Ryacudu Rd., Way Huwi, South Lampung 35365, Lampung, Indonesia; hesti.pangastuti@tp.itera.ac.id; 2 School of Food Industry, King Mongkut’s Institute of Technology Ladkrabang, Chalong Krung Rd., Lat Krabang, Bangkok 10520, Thailand; songsak.wa@kmitl.ac.th; 3 Faculty of Agro-Industry, Chiang Mai University, Chiang Mai 50100, Thailand; supatra.ka@cmu.ac.th; 4 Cluster of Innovation for Sustainable Seafood Industry and Value Chain Management, Chiang Mai University, Chiang Mai 50100, Thailand

**Keywords:** protein–polyphenol interaction, heating sequence, interfacial properties, emulsifying stability, whey protein isolate

## Abstract

Whey proteins readily form complexes with polyphenols, the structure and functionality of which are influenced by factors such as polyphenol concentration and heat treatment. However, previous studies have largely examined these factors independently, and limited information is available regarding how the sequence of heat application (pre- vs. post-complexation) interacts with varying polyphenol concentrations to modulate the structure–function relationship of whey protein-polyphenol systems. This study investigated the effects of different heating conditions and gallic acid (GA) concentration on structural and functional properties of whey protein isolate–gallic acid (WPI-GA) complexes at pH 7.0. The treatments included native whey protein isolate (WPI), preheated WPI, native WPI-GA complexes, and WPI-GA complexes at two ratios (1:0.5 and 1:1 *w*/*w*) and heated either before or after complexation. GA addition and heat treatment increased turbidity and particle size, indicating enhanced complexation. The zeta potential showed minimal change, suggesting limited involvement of electrostatic interactions. Fluorescence quenching increased with GA concentration, confirming interactions between GA and WPI. Heat treatments increased fluorescence intensity and surface hydrophobicity, likely due to protein unfolding and exposure of hydrophobic regions. Higher GA concentration enhanced antioxidant activity, reduced foaming capacity, and did not affect emulsifying properties. Preheating also decreased the foaming capacity of the complexes, whereas post-heating restored it. Both heat treatments reduced the emulsifying activity index (EAI) but increased the emulsion stability index (ESI) compared with native WPI. Overall, this study provides insight into how GA concentration and heating sequence influence the complexation and functionality of WPI, contributing to a better understanding of protein–polyphenol interactions in bioactive-enriched dairy systems.

## 1. Introduction

Whey proteins are valuable by-products obtained from the processing of cheese, Greek yogurt, and casein-based ingredients, and are widely studied in food science due to their diverse functional properties. These proteins are primarily composed of α-lactalbumin, β-lactoglobulin, bovine serum albumin, lactoferrin, and immunoglobulins [1], and are well known for their high nutritional quality and balanced amino acid composition. In addition, they contribute to improving food system performance by influencing key functional attributes such as foaming ability, emulsification, water retention, and gel formation [2]. Recently, increasing interest has been given to the interactions between whey proteins and plant-derived polyphenols. These interactions may occur through both covalent and non-covalent pathways, with hydrogen bonding, hydrophobic interactions, and van der Waals forces being the predominant non-covalent mechanisms [3]. Such complex formation has been reported to modify the structural and functional characteristics of proteins, which may in turn enhance antioxidant potential, emulsifying performance, and overall system stability [4,5,6].

Our previous study showed that the incorporation of phenolic acids into ice cream markedly affects its physicochemical and structural properties, including melting behavior, viscosity, and air-phase stability [7]. Beyond changes in the physical properties of the frozen matrix, the observed effects of phenolic acid addition suggested the presence of interactions with milk proteins, which may influence protein network formation during freezing and storage. These findings indicate that protein–polyphenol interactions play an important role in determining the functional properties of dairy-based systems containing bioactive compounds [8,9]. Based on these observations, the present study aims to gain a more fundamental understanding of the interactions between whey protein isolate (WPI) and a representative phenolic acid, gallic acid (GA). GA is a water-soluble polyphenol widely distributed in fruits, vegetables, and cereals, and has attracted considerable attention due to its strong antioxidant activity and good compatibility with aqueous food systems [10].

Previous studies have shown that heat treatment can modulate protein–polyphenol interactions by inducing conformational changes in proteins, increasing surface hydrophobicity, and facilitating sulfhydryl–disulfide exchange reactions [11,12]. Preheating is considered beneficial as it promotes controlled partial unfolding of whey proteins, thereby exposing hydrophobic and reactive groups without causing extensive aggregation or gelation. In this study, a temperature of 65 °C was selected to achieve a sub-denaturation state (approximately 28% denaturation), which is sufficient to induce partial unfolding and enhance protein reactivity toward phenolic compounds, while preserving overall structural integrity and preventing excessive aggregation or gel formation. Although this condition does not reflect conventional industrial pasteurization processes, it was intentionally applied to induce controlled conformational changes. Upon heating, whey proteins undergo partial unfolding, which increases their reactivity toward phenolic compounds and consequently influences the structural and functional properties of the final complexes. For example, preheating of WPI at 80 °C has been reported to enhance binding affinity with quercetin [13] and to strengthen EGCG-mediated interactions through combined hydrophobic and electrostatic mechanisms [14].

Although recent studies have begun to investigate the effect of heating sequence on protein–polyphenol interactions, most have focused on single processing approaches (e.g., preheating prior to complex formation) or on different protein–polyphenol systems, which limits direct comparison between heating-before and heating-after complexation. Moreover, many of these investigations have been conducted using isolated protein fractions (e.g., β-lactoglobulin), non-neutral pH conditions, or high-intensity thermal treatments that do not fully reflect typical dairy processing environments. Research specifically addressing whey protein isolate (WPI) systems at pH 7.0, which is representative of milk and dairy matrices, combined with moderate heat treatments within a pasteurization-relevant range, remains limited. In addition, although interactions between WPI and simple phenolic compounds such as gallic acid (GA) have been reported under similar conditions, systematic comparisons of how heating sequence affects both structural modifications and functional properties within a unified experimental design are still lacking.

While these studies have provided valuable insights into WPI–polyphenol interactions, the comparative effects of heating sequence—applied either before or after complex formation—on the molecular structure and functional properties of WPI-GA systems have not yet been systematically evaluated under consistent experimental conditions. Therefore, this study aims to investigate the influence of heating sequence, together with GA concentration, on the structural and functional characteristics of WPI-GA complexes under conditions relevant to dairy applications. A clearer understanding of these mechanisms will provide a scientific basis for improving protein functionality and support the rational design of protein–polyphenol systems for functional food applications, including frozen dairy products such as ice cream.

## 2. Materials and Methods

### 2.1. Material Preparation

Whey protein isolate (purity 87.6%) was purchased from Food Great Products Co., Ltd. (Bangkok, Thailand). Gallic acid (purity ≥ 99.5%) and 8-aniline-1-naphthalene sulfonate powder (purity ≥ 90%) were obtained from Sigma Aldrich (St. Louis, MO, USA). Deionized water was purchased from Winnex (Bangkok, Thailand). Sodium phosphate dibasic heptahydrate, sodium phosphate monobasic monohydrate, sodium hydroxide, 2,2-diphenyl-1-picrylhydrazyl (DPPH) (purity ≥ 90%), 2,4,6-tri(2-pyridyl-1,3,5-triazine (purity ≥ 98%), ferric chloride (purity ≥ 97%), and 2,2′-azino-bis(3-ethylbenzothiazoline-6-sulfonic acid) (ABTS) (purity ≥ 98%), Trolox standard (purity ≥ 97%), and sodium deodecyl sulfate (SDS) (purity ≥ 98%) were of analytical grade and purchased from Sigma Aldrich (St. Louis, MO, USA).

All analytical instruments used in this study, including a UV–Vis spectrophotometer (UV-1800, Shimadzu, Kyoto, Japan), a particle size analyzer (Zetasizer Nano ZS, Malvern Instruments, Malvern, UK), a fluorescence spectrometer (FluoroMax Plus, Horiba Scientific, Kyoto, Japan), and a microplate reader (EnSight™, PerkinElmer, Shelton, CT, USA), were used according to the manufacturers’ instructions.

### 2.2. Preparation of Native and Preheated Whey Protein Isolate

Native whey protein isolate (designated as “NWPI”) (0.8% *w*/*v*) was prepared by dissolving WPI in phosphate-buffered solution (PBS, 10 mM, NaH_2_PO_4_/Na_2_HPO_4_, pH 7.0) with continuous stirring for 30 min at 25 °C. The pH was adjusted to 7 using 1 M sodium hydroxide (NaOH). Preheated WPI (denoted as “HWPI”, 0.8% *w*/*v*) was prepared by dispersing WPI in phosphate-buffered solution under the same conditions, followed by heating at 65 ± 3 °C for 30 min and subsequent cooling in an ice slurry. As WPI has a denaturation temperature of ~80 °C, heating to 65 °C results in partial denaturation and unfolding of its structure [15]. This condition was specifically employed to induce controlled conformational changes, rather than to simulate typical industrial pasteurization conditions.

### 2.3. Complexation of WPI and Gallic Acid

WPI-GA colloidal complexes were prepared at ambient temperature following the method described by Li et al. [16] with slight modification. Briefly, an aqueous GA solution was mixed into an aqueous WPI solution under continuous magnetic stirring for 30 min. WPI concentration was fixed at 0.8% (*w*/*v*), while GA was added at 0.4% and 0.8% (*w*/*v*), corresponding to WPI:GA mass ratios of 1:0.5 and 1:1 (*w*/*w*), respectively. The pH of the complexes was adjusted to 7.0. Complexes formed with native WPI and GA were denoted as “NWPI-GA,” those formed with preheated WPI and GA as “HWPI-GA”, and those heated after complexation as “WPI-GAH”. For the post-complexation heat treatment, additional pH-adjusted native complex solutions were heated at 65 ± 3 °C for 30 min.

### 2.4. Turbidity Measurement

Larger particles typically result in higher turbidity due to their greater capacity to refract and scatter light [17]. To assess complex size, a spectrophotometric analysis of turbidity was conducted on the prepared WPI-GA complexes using the method described by Chima et al. [18] with absorbance measured at a wavelength of 600 nm using a spectrophotometer (UV1800, Shimadzu, Japan).

### 2.5. Zeta Potential, Particle Size, and Polydispersity Index Analysis

Zeta potential, particle size distribution, and polydispersity index of all treatments were measured at ambient temperature (25 ± 1 °C) using a Zetasizer Nano System (Z5, Malvern Instruments Ltd., Malvern, UK) following a published method [19]. Zeta potential provides insight into the surface charge of particles in suspension, which directly relates to their stability, aggregation behavior, and interaction mechanism. The polydispersity index (PdI) indicates the uniformity or heterogeneity of a particle size distribution. Prior to measurement, all samples were dissolved in sodium phosphate buffer at pH 7.0. Refractive index values of 1.46 and 1.33 were used for the protein and dispersion mediums, respectively. Particle size data were represented as distributions characterized by the intensity-weighted mean diameter (Z-average).

### 2.6. Fluorescence Spectroscopy Measurement

A fluorescence spectrophotometer (FluoroMax+ SpectroFluorometer, Horiba Scientific, Kyoto, Japan) was used to measure the intrinsic fluorescence spectra of WPI-GA complexes according to the method of Wen et al. [20]. Samples were placed in a quartz cuvette with a 1 cm optical path length. The excitation wavelength was set to 280 nm, and emission spectra were recorded in the range of 290 to 450 nm, with a constant slit width of 5 nm for both excitation and emission.

### 2.7. Surface Hydrophobicity Measurement

Surface hydrophobicity reflects the exposure of hydrophobic amino acid residues on the protein surface, which is influenced by protein structure and conformation. Changes in surface hydrophobicity can therefore be indicative of protein denaturation or complex formation. Surface hydrophobicity measurement was carried out using a fluorescence spectrophotometer according to the method of Yu et al. [21]. Sample solutions were progressively diluted to concentrations of 0.2, 0.1, 0.05, and 0.025 mg/mL using PBS (0.01 M, pH 7.0) and thoroughly mixed. Then, 20 µL of 8-aniline-1-naphthalene sulfonate solution (8.0 mmol/L, pH 7.0) was added, and the reaction was allowed to proceed in the dark for 15 min. Finally, fluorescence intensity was determined with an excitation wavelength of 390 nm and an emission wavelength of 470 nm. Surface hydrophobicity was determined as the slope of the best-fit line derived from plotting WPI concentration as the abscissa and fluorescence intensity as the ordinate.

### 2.8. Antioxidant Capacity Measurement

Antioxidant capacity was assessed with three complementary methods: 2,2-diphenyl-1-picrylhydrazyl (DPPH), ferric reducing antioxidant power (FRAP), and 2,2′-azino-bis(3-ethylbenzothiazoline-6-sulfonic acid) (ABTS). A modified DPPH assay, as described by Fan et al. [22], was used to determine free radical scavenging activity. The assay involved mixing 40 μL of sample solutions with 260 μL of 0.1 mM DPPH solution in methanol, followed by incubation in the dark at room temperature for 30 min. A Trolox calibration curve was constructed using Trolox dissolved in ethanol at concentrations ranging from 50 to 200 μg/mL. A sample blank was prepared by replacing the DPPH solution with an equal volume of water. After incubation, absorbance was measured at 517 nm. Results were expressed as Trolox equivalents (TE) per gram of sample on a dry weight basis (mg TE/g), with standard deviation (SD) reported. The FRAP assay was performed following the method of Tan et al. [22]. FRAP reagent was prepared by mixing 300 mM sodium acetate buffer, 10 mM 2,4,6-tripyridyl-s-triazine solution, and 20 mM Fe[III] chloride solution in a volumetric ratio of 10:1:1. In a 96-well microplate, 20 μL of sample or standard was combined with 280 μL of the prepared FRAP reagent. This mixture was incubated at 37 °C in the dark for 10 min, after which the absorbance was recorded at 593 nm using a microplate reader (Ensight^TM^, PerkinElmer, USA). Trolox, dissolved in absolute ethanol at concentrations ranging from 50 to 200 μg/mL, was used as the standard for quantification of FRAP radical scavenging activity. A blank was prepared by substituting the sample with water. Results were reported as milligrams of Trolox equivalents per gram (mg TE/g), averaged and with standard deviation (SD).

The ABTS decolorization assay was performed according to the method described by Tan et al. [22]. To prepare the ABTS^•+^ solution, 12.5 mL of 7 mM ABTS aqueous solution was combined with 220 μL of 140 mM potassium persulfate and incubated in the dark at room temperature for 16 h. Prior to use, the ABTS^•+^ solution was diluted with analytical-grade ethanol until its absorbance at 734 nm reached 0.7. For the assay, 10 μL of sample or standard was mixed with 290 μL of the diluted ABTS^•+^ solution in a 96-well plate. This mixture was incubated in the dark at room temperature for 6 min, after which the absorbance was measured. Trolox dissolved in absolute ethanol (50 to 500 μg/mL) served as the standard; a blank was prepared using water in place of the sample. The antioxidant capacity of each sample was calculated from the corresponding Trolox equivalent concentration obtained from the standard curve and normalized to the sample weight according to the following equation:

Antioxidant capacity (mg TE/g) = (C × V)/(1000 × m)(1)

where C is the concentration obtained from the Trolox equivalent concentration (μg/mL), V is the extraction volume (mL), and m is the sample weight (g). The results were expressed as mg Trolox equivalent (TE) per gram of sample, averaged and with SD.

### 2.9. Analysis of Foaming Properties

Foaming properties of WPI and WPI-GA complexes, namely the foaming capacity (FC) and foaming stability (FS), were measured according to a published method [23]. A 20 mL sample was transferred into a 50 mL centrifuge tube and homogenized using a high-speed homogenizer (T25 digital ULTRA-TURRAX, IKA, Staufen im Breisgau, Germany) operating at 10,000 rpm for 60 s at ambient temperature. FC was calculated from the total volume immediately after whipping, and FS from the volume after sitting undisturbed for 30 min using the following formulas:

(2) $$FC(\%)=\frac{V_{T}}{V_{o}}\times 100$$

(3) $$FS(\%)=\frac{V_{t}}{V_{o}}\times 100$$

where *V_T_* represents the total volume following high-speed agitation; *V_o_* the initial volume prior to agitation; and *V_t_* the volume after 30 min.

### 2.10. Analysis of Emulsifying Properties

Emulsifying properties were measured according to a published method [23], with specific focus on the emulsifying activity index (EAI) and emulsion stability index (ESI). To prepare mixtures, 5 mL of palm oil and 15 mL of each sample were combined in a plastic cylinder and subjected to homogenization using a high-speed homogenizer (T25 digital ULTRA-TURRAX, IKA, Germany) at a speed of 20,000 rpm for 1 min. At 0 and 10 min, 50 μL of each emulsion was pipetted out from the bottom of the cylinder and transferred into 5 mL of 0.1% (*w*/*v*) SDS solution. After mixing by vortexing, the absorbance was quantified at a wavelength of 500 nm using a spectrophotometer (UV1800, Shimadzu Corp., Japan) with a blank solution of 0.1% *w*/*v* SDS. EAI and ESI were calculated using the following formulas:

(4) $$EAI(m^{2}/g)=\frac{2\;\times \;2.303\;\times \;A_{0}\;\times \;DF}{i\emptyset C}$$

(5) $$ESI(min)=\frac{A_{0}\;\times \;10}{A_{0}-\;A_{10}}$$

where *A*_0_ and *A*_10_ represent the absorbance of the dispersion at 0 and 10 min, respectively; DF denotes the dilution factor; *i* denotes the path length of the cuvette (m); ∅ represents the oil volume fraction; and *C* represents the protein concentration (g/m^3^).

### 2.11. Statistical Analysis

Experiments were performed using three independent biological replicates (*n* = 3). Each biological replicate was measured in triplicate (technical replicates). Data are presented as mean ± standard deviation based on biological replicates. Statistical analyses consisted of one-way analysis of variance (ANOVA) followed by Tukey’s HSD test (*p* < 0.05) using IBM SPSS 23 software (Armonk, NY, USA).

## 3. Results and Discussion

### 3.1. Turbidity

**Figure 1:**
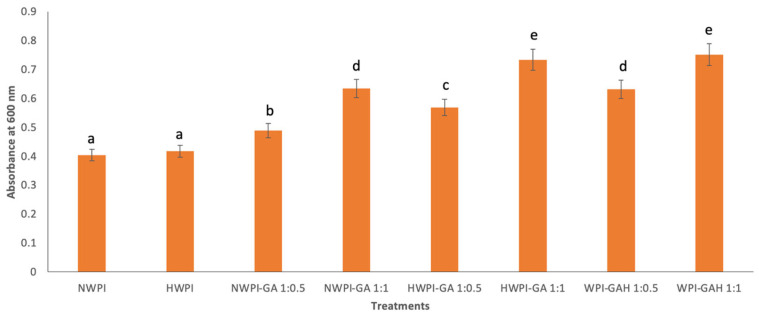
Turbidity of WPI and WPI-GA complexes (using two concentrations of GA) before and after heat treatments. Note: Abbreviations are: NWPI, native whey protein isolate; HWPI, preheated whey protein isolate; NWPI-GA, native whey protein isolate-gallic acid complex; HWPI-GA, preheated whey protein isolate-gallic acid complex; WPI-GAH, heat-treated whey protein isolate-gallic acid after complexation. Values in a column with different superscript letters are significantly different (*p* < 0.05).

Turbidity is a straightforward method for monitoring changes in aggregate size within a solution [24]. Figure 1 presents the turbidity of WPI and WPI-GA complexes before and after heat treatments. The turbidity of NWPI was 0.40 ± 0.01 and increased significantly to 0.49 ± 0.00 after complexation with GA (denoted by NWPI-GA 1:0.5), indicating the formation of complexes. A more pronounced increase was observed when more GA was added (NWPI-GA 1:1, turbidity 0.63 ± 0.02). This indicates WPI-GA interactions occur, leading to aggregate formation and the development of binding between GA and WPI. A similar trend was observed in HWPI and HWPI-GA samples, for which turbidity increased from 0.42 ± 0.00 to 0.57 ± 0.00.

Heat treatment of WPI alone did not exhibit a significant effect on turbidity. This may be attributed to the relatively limited extent of protein denaturation under the applied heating conditions. It has been reported that heating at 65 °C for 10 min results in minimal whey protein denaturation, with a denaturation degree of approximately 28.34% [25]. However, heat treatment did significantly increase the turbidity of WPI-GA complexes, with the highest turbidity observed in WPI-GAH (0.63 ± 0.02 to 0.75 ± 0.02). Heat treatment may have caused increased exposure of hydrophobic sites and induced conformational changes in WPI-GA complexes, leading to greater aggregation. These larger aggregates scatter light more effectively, resulting in higher turbidity [26].

Notably, while preheated WPI may have exposed hydrophobic side chains or altered protein conformation, it was not sufficient to produce significant aggregation. For all samples, no obvious precipitation or sedimentation was detected visually after 24 h of storage at room temperature. This indicates that the WPI-GA complexes likely formed stable colloidal particles with strong resistance to gravitational settling.

### 3.2. Zeta Potential, Particle Size, and Polydispersity Index

The surface potential of colloidal particles is an important indicator of the stability of colloidal dispersions. Table 1 presents the zeta potential, particle size, and PdI of WPI and WPI-GA complexes before and after heat treatments. Zeta potential is defined as the number of charges on particles. Wang et al. [26] demonstrated that emulsions exhibit electrostatic stability when the zeta potential is either below −30 mV or exceeds +30 mV; emulsions with values in the intervening range are more prone to agglomeration or coagulation. The NWPI dispersion exhibited a zeta potential of −8.82 ± 1.19 mV at pH 7 which is lower than commonly reported values for WPI at neutral pH (typically −20 to −30 mV)**,** and may be influenced by the ionic strength of the phosphate buffer (10 mM) as well as differences in protein source or sample conditions [27,28]. Neither complexation with GA nor heat treatment resulted in significant changes to zeta potential. It should be noted that the observed differences among treatments were not statistically significant; therefore, these variations are interpreted as indicative trends rather than definitive changes. While charge rearrangement during complexation may occur, such as the burial of charged groups within the complex interior, this cannot be conclusively inferred from the present data. The lack of significant change further suggests that electrostatic interactions may not be the dominant factor in complex formation, although this cannot be determined solely from zeta potential measurements. Heating at 65 °C is known to primarily disrupt hydrogen bonds and block van der Waals forces, which have minimal impact on zeta potential [29].

Regarding complexation itself, the native system (NWPI-GA) showed no significant effect of complex formation on particle size, but the system with preheated WPI (HWPI-GA) exhibited significantly increased particle size (see Table 1). Specifically, HWPI alone had a particle size of 146.87 ± 14.60 nm, while HWPI-GA complexes exhibited sizes of 318.84 ± 16.50 nm (1:0.5 ratio) and 344.57 ± 38.91 nm (1:1 ratio). This indicates that larger colloidal complexes were formed at higher GA concentration, and is consistent with both the turbidity analysis in this study (see Section 3.1) and a prior report by Dai et al. [17] on lactoferrin-tannic acid complexation.

Heating of whey protein isolate alone did not significantly affect its particle size; however, use of the preheated protein for complexation resulted in a significant increase in complex particle size. In particular, NWPI-GA (1:0.5) exhibited a particle size of 183.80 ± 3.84 nm, whereas HWPI-GA (1:0.5) had a particle size of 318.84 ± 16.50 nm. This indicates that preheating whey protein isolate leads to the formation of larger colloidal complexes. Greater particle size was also observed when heating was applied after complexation, with WPI-GAH consistently exhibiting larger particles compared to NWPI-GA, but not as large as HWPI-GA (*p* < 0.05 for all). Preheating of whey protein might partially unfold proteins and expose hydrophobic regions, sulfhydryl groups, and reactive amino acid residues, creating more binding sites for GA during the subsequent complexation. In contrast, when heating treatment is applied second, complexation proceeds with WPI in its native (globular) state, limiting the available binding sites for GA. The greater opportunity for initial binding and aggregation in HWPI-GA therefore leads to larger particles compared to WPI-GAH. The size results observed in this analysis are consistent with the turbidity findings (see Section 3.1).

The polydispersity index summarizes the particle size distribution of a solution. Samples in this study exhibited PdI values of 0.39 to 0.63 (Table 1), indicating relatively broad and non-ideal size distributions for values exceeding 0.4. GA has a relatively simple structure and predominantly interacts with proteins via hydrogen bonding and/or hydrophobic interactions, which can lead to the formation of uniform complexes. Statistical analysis showed all heat treatments to result in a significant reduction in complex PdI, indicating narrower size distributions for HWPI-GA and WPI-GAH complex aggregates compared to NWPI-GA.

Turbidity is closely related to particle size and dispersion, as larger or more numerous particles scatter more light, leading to higher turbidity. However, this relationship is not solely governed by particle size, but also by the internal structure and spatial organization of aggregates, which determine their light scattering efficiency. In the present study, the preheated protein-polyphenol complex sample (HWPI-GA 1:0.5) exhibited both a larger particle size and higher PdI (318.84 ± 16.50 nm, 0.44 ± 0.02) compared to the post-heated sample (WPI-GAH 1:0.5; 263.38 ± 8.42 nm, 0.42 ± 0.02). However, the preheated complex also demonstrated lower turbidity. This apparent inconsistency suggests that aggregate morphology, rather than size alone, plays a dominant role in determining optical properties.

A more detailed interpretation can be provided using fractal aggregate theory, which describes how the mass distribution and structural compactness of aggregates influence light scattering behavior. In this framework, the scattering intensity is governed not only by particle size but also by the fractal dimension, which reflects how densely primary particles are packed within an aggregate [30]. Aggregates with a more compact and dense structure (higher fractal dimension) exhibit a lower effective scattering cross-section per unit size, whereas more open and loosely connected aggregates (lower fractal dimension) scatter light more efficiently due to their extended structure.

In this study, the HWPI-GA system exhibited a less negative zeta potential (−10.70 ± 0.10 mV), indicating weaker electrostatic repulsion between particles. This condition favors closer particle-particle association, leading to the formation of denser and more compact aggregates. Consequently, despite their larger particle size, these compact aggregates scatter less light, resulting in lower turbidity.

In contrast, the WPI-GAH system showed a more negative zeta potential (−12.37 ± 0.15 mV), suggesting stronger electrostatic repulsion that limits close packing and promotes the formation of more open, branched aggregate structures. These extended aggregates possess a larger effective scattering volume and higher light scattering efficiency, which explains the higher turbidity observed despite their smaller average particle size. Nevertheless, the proposed differences in aggregate compactness and structure have not been directly visualized in this study, and further confirmation using microscopic techniques (e.g., SEM or TEM) would be required.

Similar behavior has been reported in egg white and soy protein isolate-chitosan complexes, where differences in aggregate compactness and fractal structure significantly influence turbidity and light scattering properties [31,32]. Overall, these results highlight that turbidity is governed not only by particle size but also by aggregate architecture, and they support the interpretation that preheating promotes the formation of compact aggregates, whereas post-heating leads to more open, light-scattering structures.

### 3.3. Fluorescence Spectroscopy

**Figure 2:**
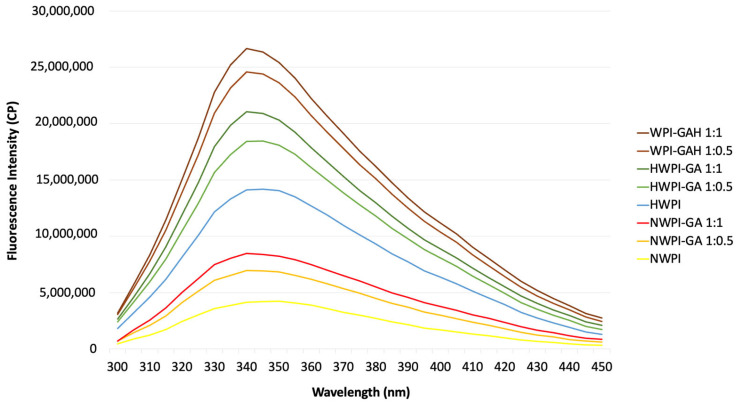
Fluorescence spectra of WPI and WPI-GA complexes (using ratios of WPI and GA 1:0.5 and 1:1) before and after heat treatments. Note: Abbreviations are: NWPI, native whey protein isolate; HWPI, preheated whey protein isolate; NWPI-GA, native whey protein isolate-gallic acid complex; HWPI-GA, preheated whey protein isolate-gallic acid complex; WPI-GAH, whey protein isolate-gallic acid complex heat-treated after complexation. The λm values were determined to be 339 nm (HWPI), 342 nm (NWPI-GA 1:0.5), 342 nm (NWPI-GA 1:1), 343 nm (HWPI-GA 1:0.5), 343 nm (HWPI-GA 1:1), 343 nm (WPI-GAH 1:0.5), and 343 nm (WPI-GAH 1:1).

The intrinsic fluorescence of proteins is caused by tryptophan (Trp) residues, the overall position of which is reflected in the fluorescence peak. Specifically, a high fluorescence intensity implies the protein to be in a folded state, with the Trp residues typically located in a hydrophobic environment. The effects of WPI-GA complexation and heat treatments on protein fluorescence are presented in Figure 2. The fluorescence emission maximum (λ_m_) of native WPI was around 339 nm. The λm values for the different treatments were determined to be approximately 339 nm (HWPI), 342 nm (NWPI-GA 1:0.5), 342 nm (NWPI-GA 1:1), 343 nm (HWPI-GA 1:0.5), 343 nm (HWPI-GA 1:1), 343 nm (WPI-GAH 1:0.5), and 343 nm (WPI-GAH 1:1), indicating a progressive shift toward longer wavelengths depending on treatment conditions. Both heating and GA addition induced a red shift (longer wavelength), indicating the microenvironment of the Trp chromophore to have changed to a more hydrophilic state. This result is consistent with other studies on whey protein-polyphenol complexes [23,33,34].

In addition to the peak shift, changes in fluorescence intensity were also observed. Quenching of WPI fluorescence by GA was evident in the obvious decrease in fluorescence intensity as GA concentration increased, both before and after heat treatment. According to Cao and Xiong [34] and Ma et al. [35], complexation with GA decreases whey protein fluorescence intensity. This quenching may be attributed to changes in the protein’s tertiary structure, which could result in tryptophan residues becoming more exposed to a polar environment. Interaction of polyphenols with the major protein fluorophores (Trp and Tyr) is also likely to occur, with all heat-treated samples showing greater fluorescence intensity compared to the corresponding native form, whether alone or complexed. These observations are consistent with previously reported heat-induced conformational changes in whey proteins, although such structural modifications were not directly confirmed in the present study [36]. In native WPI, buried intrinsic fluorophores are partially shielded within the protein’s globular structure, resulting in lower fluorescence. At 65 °C, WPI may undergo partial denaturation; this process is likely associated with conformational changes that could expose previously buried residues to the aqueous environment, thereby increasing their accessibility to excitation and resulting in higher fluorescence intensity. With regard to different heat treatments, the WPI-GAH sample exhibited slightly lower fluorescence intensity than the HWPI-GA sample.

### 3.4. Surface Hydrophobicity

**Figure 3:**
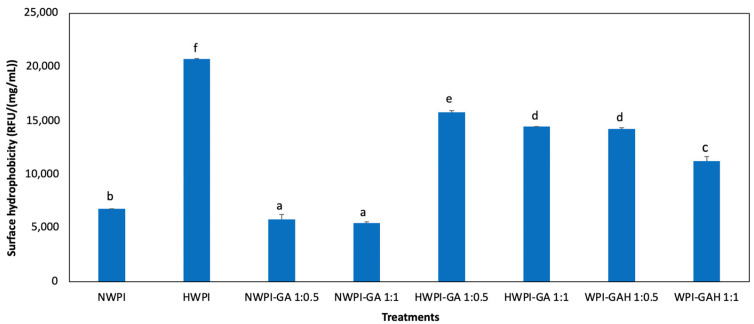
Surface hydrophobicity of WPI and WPI-GA complexes (using two concentrations of GA) before and after heat treatments. Note: Abbreviations are: NWPI, native whey protein isolate; HWPI, preheated whey protein isolate; NWPI-GA, native whey protein isolate-gallic acid complex; HWPI-GA, preheated whey protein isolate-gallic acid complex; WPI-GAH, whey protein isolate-gallic acid complex heat-treated after complexation. Bars with different superscript letters are significantly different (*p* < 0.05).

The surface hydrophobicity of a protein reflects the number of hydrophobic groups exposed on its surface when in contact with a polar solvent. Here, complexation with GA was found to decrease WPI surface hydrophobicity, whereas heat treatment consistently increased it (Figure 3). In specific terms, NWPI-GA (1:0.5) exhibited a surface hydrophobicity of 5792.17 RFU/(mg/mL), whereas that of NWPI alone was 6778.43 RFU/(mg/mL); a similar reduction was observed in HWPI-GA compared to HWPI. This decrease is related to the burial of hydrophobic amino acid residues within the protein during complexation [37]. Meanwhile, HWPI exhibited the highest surface hydrophobicity among all tested samples. This is attributed to heat-induced structural changes that result in exposure of hydrophobic regions. Heat treatment of whey proteins causes partial unfolding of tertiary and secondary structure, which destabilizes their native conformation and exposes previously buried hydrophobic residues [38,39].

Heating of proteins may be associated with disulfide bond rearrangement and the formation of non-covalent interactions (e.g., hydrophobic or hydrogen bonding) that can contribute to the stabilization of partially unfolded structures. Such structural changes may increase the protein’s ability to interact with the hydrophobic probes used in surface hydrophobicity assays. Correspondingly, disruption of intramolecular interactions within the native protein may allow more hydrophobic groups to interact with the surrounding aqueous environment.

Heat treatment was also observed to affect the surface hydrophobicity of WPI when complexed with GA. Specifically, the hydrophobicity of NWPI-GA was 5792 RFU/(mg/mL), which increased to 15,779.30 RFU/(mg/mL) for HWPI-GA and 14,237.91 RFU/(mg/mL) for WPI-GAH. This result aligns with previous studies, such as on mung bean globulin-polyphenol and whey protein isolate-anthocyanin complexes, that observed complexes to have higher surface hydrophobicity compared to their native protein [21,35]. Interestingly, WPI-GAH exhibited lower surface hydrophobicity compared to HWPI-GA. In HWPI-GA, preheating of WPI may induce partial unfolding or conformational rearrangement, potentially exposing hydrophobic regions before GA is introduced. These exposed hydrophobic regions may remain more accessible for interaction, leading to higher surface hydrophobicity in the final complex. In contrast, for WPI-GAH, the binding of GA to WPI occurs prior to heating. This interaction may partially mask or stabilize certain regions of the protein structure, thereby limiting further exposure of hydrophobic groups during subsequent heating, resulting in relatively lower surface hydrophobicity. It should be noted that these interpretations are based on indirect physicochemical measurements and are therefore indicative rather than definitive. Additional structural analyses employing CD, FTIR, and SDS-PAGE will be necessary to distinguish between covalent and non-covalent binding mechanisms, as well as to better interpret the difference between HWPI-GA and WPI-GAH systems.

### 3.5. Antioxidant Properties

To further explore the effects of GA addition and heating on whey protein, antioxidant capacity was evaluated using DPPH and ABTS radical scavenging assays, as well as the FRAP assay for reducing power. The antioxidant activity reported in this study reflects the combined effects of free and bound GA, as no separation step was applied to distinguish between these fractions. Results are presented in Table 2. The antioxidant activity exhibited by NWPI is attributed to the presence of sulfhydryl groups specifically in amino acids of lactoferrin, such as Met and Cys, which can act as antioxidants during reduction reactions [40]. Aromatic amino acids in lactoferrin can likewise react with DPPH and ABTS radicals [41].

Complexation with GA was observed to significantly enhance radical scavenging activity as measured by both DPPH and ABTS assays, with antioxidant capacity increasing in a concentration-dependent manner. This improvement is primarily attributed to the phenolic hydroxyl groups present in GA, which act as hydrogen donors to neutralize free radicals, thereby boosting the overall antioxidant potential [42].

Interestingly, differing trends in radical scavenging activity were evident depending on the treatment. Preheating the whey protein prior to complexation (HWPI-GA samples) did not significantly alter antioxidant properties. Residual-free GA may have contributed to the antioxidant activity observed for NWPI-GA samples, since the preheating exposes more binding sites and therefore HWPI-GA samples feature more GA binding than NWPI-GA samples. Although preheating may expose additional binding sites and enhance GA–protein interactions, the comparable antioxidant capacity observed between NWPI-GA 1:1 and HWPI-GA 1:1 samples suggests that increased binding does not necessarily translate into higher measured antioxidant activity. This may be attributed to the fact that antioxidant assays such as DPPH, ABTS, and FRAP primarily detect free or accessible phenolic hydroxyl groups. Consequently, GA that is more strongly bound within the protein matrix in HWPI-GA systems may be less accessible for radical scavenging reactions, resulting in similar antioxidant values. Additionally, residual unbound GA in NWPI-GA systems may compensate for the lower degree of complexation, contributing to the overall antioxidant capacity. This was likely a result of thermal degradation of GA, which reduces the availability of functional hydroxyl groups and thereby reduces radical scavenging efficacy. However, since the antioxidant activity of free GA was not directly measured, the extent to which the observed change is specifically attributable to protein-polyphenol complexation remains unclear. Future studies incorporating separation techniques (e.g., ultrafiltration or dialysis) will therefore be necessary to quantify free and bound GA and to more accurately assess the antioxidant contribution of the complexes.

### 3.6. Foaming Properties

The determination of foaming properties revealed NWPI-GA complexes to have lower foaming capacity (168.33% ± 2.89) in comparison to NWPI alone (187.50% ± 12.50) (Table 3). The same trend was observed for heat-treated protein and corresponding complexes. Together, these findings suggest the incorporation of GA to inhibit the ability of protein molecules to promote stable air bubble formation. Foaming properties of proteins are related to numerous factors, including surface hydrophobicity, surface tension, conformation, protein–protein interaction, and aggregation formation [43]. This particular effect may be due to the increasing size of WPI-GA complexes with GA concentration (see Section 3.2), which likely hindered their movement through the aqueous phase to the air–water interface [44].

Heating treatment also had significant effects on foaming capacity, with preheated HWPI-GA exhibiting significantly reduced capacity compared to NWPI-GA (150.00% ± 5.00 versus 168.33% ± 2.89). Preheating likely causes partial denaturation of WPI, exposing more hydrophobic groups, potentially producing bigger complexes with correspondingly reduced surface activity, and ultimately making the complexes less able to migrate to the air-water interface and form a stable film. This finding is similar to reports on corn protein hydrolysate-tannic acid complexes [45,46], which exhibited 16% lower foaming capacity compared to protein alone. Heat treatment after complexation (WPI-GAH) achieved better foaming capacity than did pretreatment, remaining similar to the native protein. Heating the WPI-GA complexes likely results in smaller, more dispersed aggregates that have sufficient flexibility to enhance foaming ability (see Section 3.2). Foaming stability was not impacted by either the addition of GA or the application of heat, whether before or after complexation.

Collectively, the present data support the findings of our previous study [7], which reported that GA can reduce the foaming properties of ice cream mix. Since ice cream mix undergoes heat treatment, these results suggest that GA concentration directly affects its foaming ability, with more GA leading to decreased foaming. In this study, the foaming tests conducted were designed to evaluate the system’s ability to incorporate and retain air, which are key indicators of functional performance. Although overrun was not measured directly, foaming capacity and foam stability provide meaningful insight into the air-holding behavior of the WPI-GA complexes. These measurements reflect how effectively the system can generate foam and maintain its structure over time, thereby serving as suitable proxies for overall foam performance within the scope of the present experimental design.

### 3.7. Emulsifying Properties

The emulsifying activity index (EAI) is a key property that measures the ability of a protein to adsorb at the oil-water interface [41], while the emulsifying stability index (ESI) assesses the ability of an emulsion to stay dispersed and resist aggregation or flocculation [42]. Generally, protein adsorption at the oil–water interface is a physical phenomenon, and conformational changes in proteins affect their capacity for such adsorption [43]. In this study, HWPI exhibited higher EAI and ESI (EAI = 28.73 ± 2.85 m^2^/g; ESI = 24.49 ± 2.20 min) compared to NWPI (Table 3), indicating that preheating of protein by itself induced flexibility, fostering good emulsifying activity and stability. Heat treatment of WPI induces partial unfolding, exposing hydrophobic regions that interact more effectively with oil droplets in emulsions, hence the improved ability to adsorb at oil-water interfaces and enhanced emulsifying capacity. In addition, the formation of a cohesive interfacial protein layer during preheating can lead to increased emulsion stability by preventing coalescence of oil droplets [44].

Complexation of native WPI with GA did not significantly affect EAI and ESI compared to the control (NWPI) (EAI = 25.77 ± 1.80 versus 24.97 ± 0.73 m^2^/g; ESI = 15.99 ± 0.77 versus 14.78 ± 0.52 min). However, heat treatment resulted in lower EAI but higher ESI compared to NWPI, regardless of whether heating occurred before or after complexation. Thus, both heat treatments exhibited a similar shift in emulsifying properties.

The reduced emulsifying capacity of heat-treated WPI-GA complexes may be associated with conformational changes in the whey protein, which could expose additional binding sites for GA. This increased complexation led to larger particles, which have less flexibility and effectiveness as emulsifiers. These factors reduce the protein’s ability to efficiently form new oil droplet interfaces during emulsification [45].

At the same time, heat treatment and whey protein-GA interactions both enhanced emulsion stability. Heat-induced aggregates and polyphenol binding create stronger, more cohesive interfacial films, improving droplet resistance to coalescence and reducing disruption over time. The increased surface hydrophobicity from heat treatment also strengthens the interaction between proteins and oil droplets, contributing to a higher ESI [46].

The relationship between foaming and emulsifying properties can be explained by their common dependence on protein adsorption at interfaces, although the specific interfacial requirements differ. In this study, complexation of WPI with GA resulted in a reduction in foaming capacity compared to both native WPI and preheated WPI alone, with the lowest foaming capacity observed in samples where WPI was preheated prior to complexation. This behavior is consistent with the reduced EAI observed in heat-treated WPI-GA complexes, suggesting that the formation of larger aggregates during heating and complexation hinders the ability of proteins to rapidly adsorb and form new interfaces. However, in contrast to foaming capacity and EAI, the emulsifying stability index (ESI) increased following complexation and heat treatment. This indicates that although interfacial adsorption is impaired, the resulting interfacial films are stronger and more cohesive.

These findings highlight that while both foaming and emulsifying properties rely on interfacial activity, they respond differently to structural modifications. Reduced molecular flexibility and increased aggregation negatively affect the formation of new interfaces (bubble formation and emulsifying activity), whereas enhanced intermolecular interactions and network formation at the interface contribute to improved stability (foam or emulsion stability).

## 4. Conclusions

This study demonstrated that GA addition and heat treatment markedly influenced the structural and functional properties of WPI-GA complexes. Complex formation resulted in a clear decrease in fluorescence intensity and surface hydrophobicity (from 6778 to 5792 RFU/(mg/mL), approximately 15% reduction), confirming the occurrence of protein–polyphenol interactions. In contrast, subsequent heat treatment significantly increased these properties (from 5792 to 15,779 RFU/(mg/mL), approximately 2.7-fold increase), likely due to heat-induced conformational changes and enhanced exposure of hydrophobic regions.

Preheating of WPI prior to complexation (HWPI-GA) increased surface hydrophobicity, whereas post-heating after complexation (WPI-GAH) reduced foaming capacity but enhanced antioxidant activity (from 261.76 to 289.69 mg TE/g sample). The addition of GA markedly improved antioxidant capacity, reaching up to 7.6-fold higher than that of WPI alone; however, higher GA concentrations decreased foaming capacity without significantly affecting emulsifying activity. Both pre- and post-heating treatments decreased the emulsifying activity index (EAI) but increased the emulsion stability index (ESI), suggesting improved emulsion stability despite reduced interfacial adsorption efficiency.

Differences in turbidity and particle size among the treatments suggest that light scattering behavior is influenced more by aggregate structure than by particle size alone. Preheating likely promotes the formation of more compact aggregates, whereas post-heating appears to produce more open aggregate structures. From an application perspective, HWPI-GA systems may be more suitable for enhancing antioxidant functionality, while WPI-GAH systems may be better suited for applications requiring improved foaming performance and emulsion stability.

Overall, these findings provide quantitative insights into protein–polyphenol interactions and highlight practical strategies for tailoring functional properties in bioactive-enriched food systems. Further studies are warranted to elucidate the effects of protein–polyphenol complex formation, with particular emphasis on selecting polyphenolic compounds that are more relevant and commonly present in food systems.

## Figures and Tables

**Table 1:** Zeta potential, particle size, and polydispersity index (PdI) of WPI and WPI-GA complexes (using two concentrations of GA) before and after heat treatments.

Treatments	Zeta Potential (mV) ^ns^	Particle Size (nm)	PdI
NWPI	−8.82 ± 1.19	171.30 ^ab^ ± 8.91	0.64 ^b^ ± 0.13
HWPI	−10.32 ± 0.59	146.87 ^a^ ± 14.60	0.62 ^b^ ± 0.09
NWPI-GA 1:0.5	−10.10 ± 0.10	183.80 ^b^ ± 3.84	0.62 ^b^ ± 0.01
NWPI-GA 1:1	−11.53 ± 1.72	207.97 ^b^ ± 14.09	0.63 ^b^ ± 0.10
HWPI-GA 1:0.5	−10.70 ± 0.10	318.84 ^d^ ± 16.50	0.44 ^a^ ± 0.02
HWPI-GA 1:1	−11.80 ± 1.42	344.57 ^d^ ± 38.91	0.42 ^a^ ± 0.03
WPI-GAH 1:0.5	−12.37 ± 0.15	263.38 ^c^ ± 8.42	0.42 ^a^ ± 0.02
WPI-GAH 1:1	−13.57 ± 0.81	271.03 ^c^ ± 3.43	0.39 ^a^ ± 0.03

*Note*: Abbreviations are: NWPI, native whey protein isolate; HWPI, preheated whey protein isolate; NWPI-GA, native whey protein isolate-gallic acid complex; HWPI-GA, preheated whey protein isolate-gallic acid complex; WPI-GAH, whey protein isolate-gallic acid complex heat-treated after complexation. Values are given as means ± standard deviation. Values in a column with different superscript letters are significantly different (*p* < 0.05). ns = Not significant.

**Table 2:** Antioxidant properties of WPI and WPI-GA complexes (using two concentrations of GA) before and after heat treatments.

Treatments	DPPH Scavenging (mg TE/g)	ABTS Scavenging (mg TE/g)	FRAP Scavenging (mg TE/g)
NWPI	41.56 ± 2.50 ^a^	114.2 ± 4.73 ^a^	37.23 ± 2.04 ^a^
HWPI	40.53 ± 1.17 ^a^	114.76 ± 4.29 ^a^	32.20 ± 3.53 ^a^
NWPI-GA 1:0.5	261.76 ± 6.31 ^c^	273.77 ± 2.97 ^c^	257.73 ± 6.28 ^c^
NWPI-GA 1:1	303.50 ± 6.85 ^e^	315.57 ± 3.36 ^e^	297.83 ± 7.49 ^e^
HWPI-GA 1:0.5	255.66 ± 2.94 ^c^	275.70 ± 3.93 ^c^	251.66 ± 2.85 ^c^
HWPI-GA 1:1	299.38 ± 5.95 ^e^	312.66 ± 3.30 ^e^	293.18 ± 9.11 ^e^
WPI-GAH 1:0.5	246.69 ± 2.63 ^b^	260.21 ± 7.07 ^b^	241.68 ± 3.56 ^b^
WPI-GAH 1:1	289.69 ± 2.05 ^d^	304.58 ± 3.29 ^d^	284.03 ± 2.78 ^d^

*Note:* Abbreviations are: NWPI, native whey protein isolate; HWPI, preheated whey protein isolate; NWPI-GA, native whey protein isolate-gallic acid complex; HWPI-GA, preheated whey protein isolate-gallic acid complex; WPI-GAH, whey protein isolate-gallic acid complex heat-treated after complexation. Results were expressed as Trolox equivalents (TE) per gram of sample on a dry weight basis (mg TE/g). Values are given as means ± standard deviation. Values in a column with different superscript letters are significantly different (*p* < 0.05).

**Table 3:** Foaming and emulsifying properties of WPI and WPI-GA complexes (using two concentrations of GA) with before and after heat treatments.

Treatments	Foaming Capacity (%)	Foaming Stability (%) ^ns^	Emulsifying Activity Index (m^2^/g)	Emulsifying Stability Index (min)
NWPI	187.50 ± 12.50 ^d^	92.54 ± 2.25	24.97 ± 0.73 ^c^	14.78 ± 0.52 ^a^
HWPI	185.00 ± 10.00 ^d^	91.05 ± 1.62	28.73 ± 2.85 ^d^	24.49 ± 2.20 ^e^
NWPI-GA 1:0.5	168.33 ± 2.89 ^c^	93.08 ± 1.65	25.77 ± 1.80 ^c^	15.99 ± 0.77 ^ab^
NWPI-GA 1:1	165.00 ± 10.00 ^bc^	92.98 ± 2.13	25.63 ± 1.64 ^c^	16.06 ± 1.24 ^ab^
HWPI-GA 1:0.5	150.00 ± 5.00 ^ab^	93.33 ± 0.22	18.53 ± 0.42 ^b^	17.80 ± 0.44 ^bc^
HWPI-GA 1:1	139.17 ± 10.10 ^a^	95.94 ± 4.17	11.92 ± 0.63 ^a^	20.69 ± 1.92 ^d^
WPI-GAH 1:0.5	173.33 ± 2.89 ^cd^	93.28 ± 1.60	17.55 ± 0.36 ^b^	17.21 ± 0.22 ^bc^
WPI-GAH 1:1	151.67 ± 12.58 ^ab^	92.59 ± 5.23	13.35 ± 1.20 ^a^	18.42 ± 1.07 ^c^

*Note:* Abbreviations are: NWPI, native whey protein isolate; HWPI, preheated whey protein isolate; NWPI-GA, native whey protein isolate-gallic acid complex; HWPI-GA, preheated whey protein isolate-gallic acid complex; WPI-GAH, whey protein isolate-gallic acid complex heat-treated after complexation. Values are given as means ± standard deviation. Values in a column with different superscript letters are significantly different (*p* < 0.05). ns = Not significant.

## Data Availability

The original contributions presented in this study are included in the article. Further inquiries can be directed to the corresponding author.

## Abbreviations

The following abbreviations are used in this manuscript:

WPI	Whey Protein Isolate
GA	Gallic acid
NWPI	Native Whey Protein Isolate
HWPI	Preheated Whey Protein Isolate
